# Native-mimicking in vitro microenvironment: an elusive and seductive future for tumor modeling and tissue engineering

**DOI:** 10.1186/s13036-018-0114-7

**Published:** 2018-09-12

**Authors:** Girdhari Rijal, Weimin Li

**Affiliations:** Department of Biomedical Sciences, Elson S. Floyd College of Medicine, Washington State University, Spokane, WA 99210 USA

**Keywords:** ECM, 3D cell culture, Native tissue, Biomaterial, Scaffold, Hydrogel, Microenvironment, Tumor modeling, Tissue engineering, Regeneration

## Abstract

Human connective tissues are complex physiological microenvironments favorable for optimal survival, function, growth, proliferation, differentiation, migration, and death of tissue cells. Mimicking native tissue microenvironment using various three-dimensional (3D) tissue culture systems in vitro has been explored for decades, with great advances being achieved recently at material, design and application levels. These achievements are based on improved understandings about the functionalities of various tissue cells, the biocompatibility and biodegradability of scaffolding materials, the biologically functional factors within native tissues, and the pathophysiological conditions of native tissue microenvironments. Here we discuss these continuously evolving physical aspects of tissue microenvironment important for human disease modeling, with a focus on tumors, as well as for tissue repair and regeneration. The combined information about human tissue spaces reflects the necessities of considerations when configuring spatial microenvironments in vitro with native fidelity to culture cells and regenerate tissues that are beyond the formats of 2D and 3D cultures. It is important to associate tissue-specific cells with specific tissues and microenvironments therein for a better understanding of human biology and disease conditions and for the development of novel approaches to treat human diseases.

## Background

Native microenvironment (NME) of live tissue is a mechanophysiological space provided to tissue cells, which in turn contribute to the overall appearance and function of the tissue. Because of the versatility and heterogeneity of human tissues and their specific organizations in organs, it is often difficult to precisely define a tissue NME. Thus, NME is rather specified on the basis of physical, physiological, metabolic and other functions of particular tissues or organs. For example, the bone microenvironment is necessary for normal growth and resorption of bone tissues while the heart microenvironment is essential for cardiomyocytes, other heart cells and blood vessels to maintain the heart muscle kinetic functions. Normal NME therefore plays vital roles in maintaining the integrity and functionality of tissues ranging from growth to resorption and static to kinetic activities, with an exception in regenerative microenvironment (RME), where a reprogrammed tissue growth is involved.

Intracellular, intercellular and extracellular spaces and components comprise the foundation of microenvironments under native conditions, which comprehensively include the spatial arrangement and distribution of different types of cells as well as their functionally coordinating intra- and extra-cellular physical and signaling networks, the structural and mechanical properties of extracellular matrix (ECM), the temperature, the pH, the partial pressure of O_2_ and CO_2_ within the interstitial space, etc. Tumor microenvironment (TME) is an abnormal native physiological condition, where tumor cells and their associated stromal cells undergo uncontrolled growth, proliferation, migration, excessive deposition of certain extracellular proteins and other cancerous cellular activities that result in irregular ECM networks and tissue growth [1, 2].With our accumulating knowledge about ECM, tissue cells and their associated regulating factors under pathophysiological conditions [3, 4], encouraging advances in the fields of biomedical and bioengineering research have been achieved by means of the use of various scaffolding materials and techniques for spatial tissue culture as well as for tissue repair and regeneration. These advances have brought about close mimicry of specific tissue microenvironments for more precise modeling of human disease conditions such as breast cancer compared to traditional 2D tissue cultures [5–7]. Importantly, it has been realized that a disease condition within a local tissue microenvironment is the nidus related to a global systemic change [8].

Here we focus on summarizing and discussing the major cells within human connective tissues, the mostly used scaffolding materials to mimic tissue ECMs for spatial cell cultures, certain tissue-associated chemokines, growth factors (GFs) and hormones, and physiological conditions such as temperature, pH and air gas levels in tissues. The purpose of this review is to better understand the roles of the major factors essential for the maintenance of native microenvironment and to utilize these factors in applications of creating native-like microenvironments in in vitro culture systems for advanced modeling of human diseases and tissues.

### Cells of native microenvironment

**Fig. 1:**
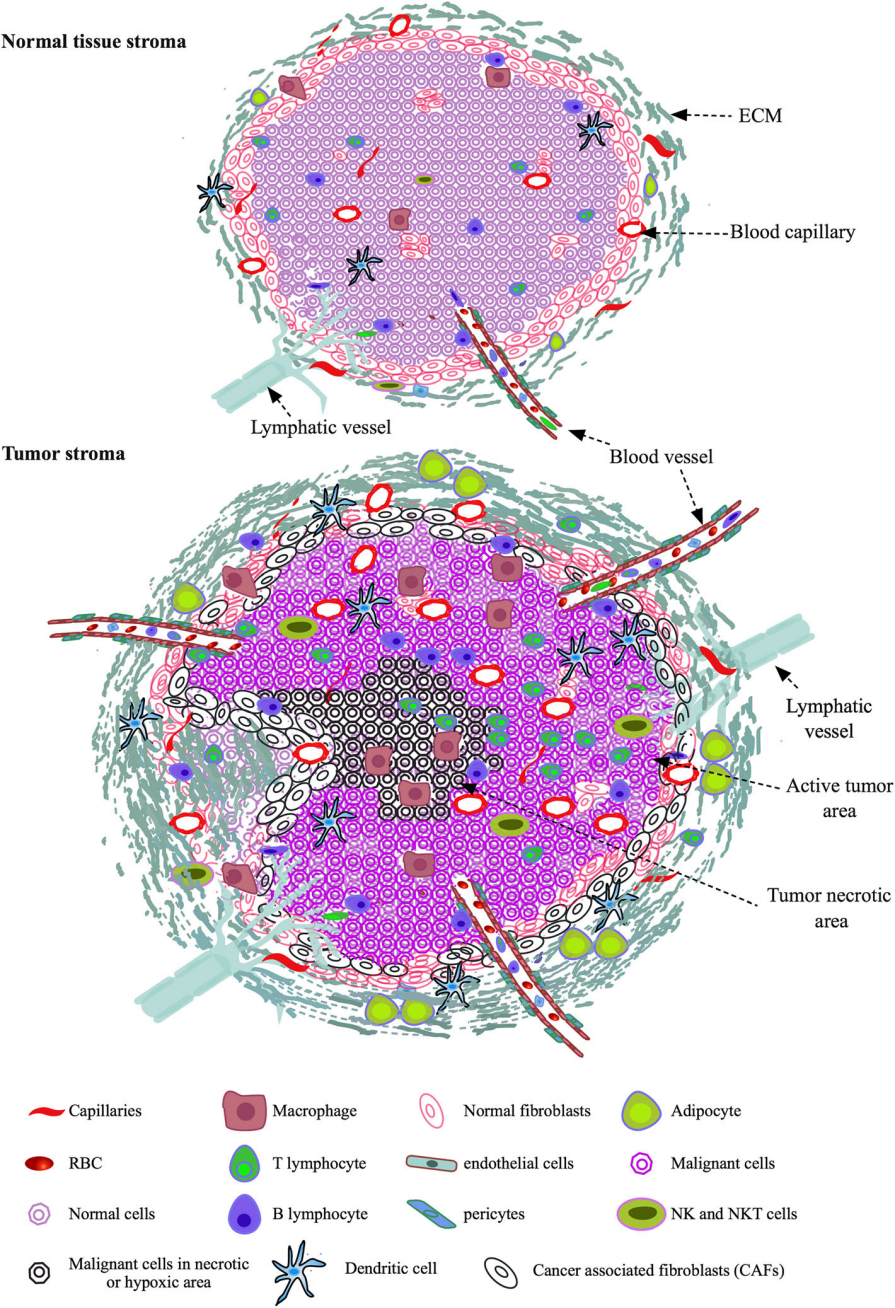
Normal and tumor tissue stroma. Normal tissue stroma shows normal pattern of cell and ECM organizations with minimal distribution of immune cells and regular supply of oxygen and nutrients through blood vessels and capillaries. Tumor tissue stroma is more complex and rich in cell and ECM contents with irregular organization compared to normal tissue stroma. High infiltration of immune cells, cancer cells and CAFs/TAFs and increased ECM protein deposition in tissue stroma is characteristic of tumor microenvironment. Tumor activated area is perfused with high amount of blood supply whereas tumor necrotic area is lack of blood supply. Adipocytes in tumor stroma provide additional energy to the cells living in the microenvironment and actively participate in tumor progression

Most of the human connective tissues contain tissue specific cells, cells of vasculature, lymphatic and immune system along with other cells such as migrating stem cells, fibroblasts, pericytes, and tissue associated adipocytes (Fig. 1). These cells are embedded within the interwoven fibrillar structures of ECM lattices that are filled with interstitial amorphous ground substance and fluid. Thus, tissue cells live in spatial and interactive microenvironments.

#### Tissue specific stem cells

Tissue specific stem cells are specified somatic or adult stem cells or mesenchymal cells, which have potentials to differentiate into different types of cells in specific tissues or organs, for example myoepithelial stem cells for glandular epithelium [9] and hematopoietic stem cells for various blood cells [10]. Some tissues or organs have tissue specific stem cells, which are able to regenerate and repair damaged tissues [11]. Breast-specific spindle-shaped myoepithelial cells, which line outside luminal epithelial cells and away from mammary gland ducts, adhere to basement membrane (BM) via hemidesmosomes and to adjacent luminal epithelial and myoepithelial cells by desmosomes [9, 12]. Cytokeratins (CK) such as CK5, CK14 and CK17 maintain the integrity of myoepithelial cells and support their attachment to BM and adjacent cells [13]. The cytoplasma of myoepithelial cells is filled with different types of functional proteins such as actin, myosin, fibronectin, collagen, nidogen, activin and laminin [14, 15]. The membranes of myoepithelial cells possess receptors, which include integrins, particularly β4 and α1, and E-cadherins that mediate cell-matrix and cell-cell interactions [16]. Furthermore, myoepithelial cells produce BM proteins such as laminin-1, laminin-5, collagen IV, fibronectin, and a number of tumor suppressor proteins including p63, p73, 14–3-3 sigma and maspin. Expression of morphogens and certain GFs in a coordinated manner during morphogenesis of myoepithelial cells helps maintain the correct polarity of luminal epithelial cells. Myoepithelial cells may have hierarchical differentiation pattern among myoepithelial lineages with expression of different types and levels of certain proteins. Together with BM, myoepithelial cells act as a natural barrier with selective permeability to small molecules and tumor suppressors, physically preventing cancer cell invasion and functionally suppressing tumor growth by releasing proteinase and angiogenic inhibitors [17, 18]. However, myoepithelial cells profoundly contribute to the maintenance of TME for tumor progression through their roles in paracrine signaling by expressing extracellular proteins, various chemokines, angiogenic factors and GFs that remodel BM in favor of the colony expansion of cancer cells. Because of these functionalities, myoepithelial cells are also involved in regulation of the progression of ductal carcinoma in situ (DCIS) to invasive breast cancer [17, 19]. Furthermore, myoepithelial cells can be triggered by tumor cells for the expression of invasion-associated molecules such as tenascin to promote tumor invasion and growth [20].

#### Migrating stem cells

Stem cells have an innate migrating ability as exhibited during embryogenesis, where they can invade tissues and migrate remotely for the formation of tissues [21]. Natural suppression of the migrating ability of stem cells after embryogenesis is necessary to maintain the NME of tissues. However, the migrating ability of stem cells has been shown to reappear in certain tissues when NME is altered due to epithelial-mesenchymal transition (EMT) and changes in cytoskeleton structure, cell polarity and ECM [22]. The migrating stem cells maintain normal NME by migrating toward tissue injury sites and repairing damaged tissues [23]. Sometimes, NME cannot be restored during tissue repairing process because of DNA mutation-induced conversion of migrating stem cells to cancer stem cells (CSCs), leading to the formation of TME [24, 25]. TME further enhances CSCs for local tumor growth and metastasis [26]. CSCs are heterogeneous in nature, serving as sources for progenitor cancer cells without self-renewal ability or differentiated cancer cells with self-renewal ability [27].

#### Endothelial cells

Endothelial cells are highly specialized and their functions vary considerably from one type of tissue to another. For example, vascular endothelial cells in blood-brain barrier restrict the passage of most molecules into the brain, whereas those in fenestrated capillary tuft of kidney glomerulus filter molecules required by the tissue. Irrespective of certain specific functions, endothelial cells are involved hierarchically in forming blood vessels that transport oxygen, nutrients, and various factors throughout the body. Stability and contractility of large blood vessels are provided by smooth muscle cells (SMCs) that wrap around the endothelial lining, whereas final capillaries are surrounded by pericytes, the perivascular cells that provide structural support to capillary endothelial cells in the microvasculature [28]. Angiogenic factors such as vascular endothelial GFs (VEGFs), fibroblast GFs (FGFs), platelet-derived GFs (PDGFs) and chemokines stimulate endothelial cells and pericytes to form new blood vessels and repair damaged vessels to maintain NME in tissues. Abnormal and excessive angiogenic signals either from inflammatory or malignant cells to the quiescent endothelial cells lead to neovascularization that is needed for TME and tumor growth [29]. The tumor vasculature in TME is abnormal with rapid turnover in its structures and functions, including chaotic heterogeneous branching and uneven leaky vessel lumen that increase interstitial fluid pressure and facilitate tumor cell migration [30]. Lymphatic endothelial cells can also form excessive vessel sprouting in TME lymphatic tissues under pathological conditions, such as lymphatic hyperplasia, through expressing high levels of VEGFC or VEGFD and altering immune responses to affect cancer progression [31, 32].

#### Fibroblasts

Fibroblasts are the most common but least specialized connective tissue cells that exist in connective tissues throughout the body. They are morphologically heterogeneous with versatile appearance depending on tissues, organs, and the activities of the cells [33, 34]. In NME, the main function of fibroblasts is to maintain architectural integrity of connective tissues by depositing ECM proteins like collagens, glycoproteins, proteoglycans, and laminin. During tissue repairing process in NME, fibroblasts within the tissue get activated, proliferate, migrate towards the injured site, and produce ECM to heal the injury [35]. After the tissue damage is repair, the activities of the fibroblasts decrease and the cells remain minimally active with normal phenotypes [36].

Fibroblasts in TME, generally known as tumor associated fibroblasts (TAFs), are activated fibroblasts that undergo various biological and morphological transition in response to tumor progression. They are one of the major components of tumor stroma with pleiotropic actions on tumors and play important roles in maintaining an optimal TME for cancer cell survival and proliferation [37, 38]. They get activated perpetually without reverting to their normal activity and phenotype, and can withstand severe stress without undergoing apoptosis that is usually lethal to most of other cells [39]. Tumor is always associated with TAFs that produce different biomolecules, support cancer cell transformation, induce local inflammation and angiogenesis to promote tumor growth and metastasis [39, 40].

#### Adipocytes

Adipocytes are stromal cells that normally present in fat-associated connective tissue stroma. Apart from an energy storage, they produce hormones, GFs, chemokines and other cytokines in NME [41]. In tumors, adipocytes form tumor stroma along with cancer cells, fibroblasts and other stromal cells in TME [42]. It was shown that early stage cancer cell growth and invasion occur in close proximity to adipocytes [43]. Adipocytes promote cancer invasion by releasing excessive amount of adipokines, cytokines, collagen IV and inducing production of matrix metalloproteinases (MMPs) in TME for cancer cell migration [41, 43, 44]. In addition, adipocytes provide fatty acids as fuel for the metabolic needs of cancer cells [45].

#### Immune cells

Immune cells include both granulocytes (neutrophils, eosinophils and basophils) and agranulocytes (lymphocytes and macrophages). Neutrophils are granulocytes that account for 50–70% of all leukocytes and responsible for counteracting acute infection. Their maturation depends on various stimulating factors including the granulocyte-colony stimulating factor (G-CSF) and the granulocyte-macrophage-colony stimulating factor (GM-CSF). The release of neutrophils to blood stream from bone marrow depends upon various triggering factors such as IL-23, IL-17, G-CSF and other chemokines [46]. Neutrophils are attracted by various ligands including CXCL1, CXCL2, CXCL5 to tumor site and play central roles as tumor-associated neutrophil (TAN) in tumor inflammation and development from initiation to metastasis [47, 48].

Different lymphocytes and their subpopulations exist within human normal tissue stroma that protect the NME of tissues from pathogens, injuries and other tissue damages. Among T lymphocytes, cytotoxic memory T cells (CD8^+^CD45RO^+^) are normally antigen responsive, capable of killing tumors cells and are therefore strongly associated with good prognosis of cancers [49]. CD4^+^ T helper cells (Th1), which produce cytokines like interleukins-2 (IL-2) and interferon gamma (IFN-γ), support CD8^+^ T (Th8) cells. Although, most of the lymphocytes are associated with good prognosis, some Th2, Th17 and B cells promote tumor growth by releasing interleukins and suppressing immune responsive regulatory T cells. Moreover, T cells produce transforming growth factor beta (TGF-β) and IL-10, are involved in cell-mediated contact through cytotoxic T-lymphocyte antigen 4 (CTLA4), and are also characterized by expressing forkhead box P3 (FOXP3) transcription factor and the T cell activation marker CD25 [50, 51]. It was proposed that early response of a tissue to a neoplasm is similar to its response to an acute injury, and failure to resolve the injury leads to chronic inflammation that is prone to early cancer development [52]. Since high level of regulatory T cells in TME was associated with bad prognosis in some cancers [53, 54], it was suggested to be one of the hallmarks of TME and cancer development [55]. Both natural killer (NK) and natural killer T (NKT) cells are also lymphocytes that are able to kill transformed cancer cells or viruses. When stimulated, NK and NKT release cytokines such as IL-2, IL-12 and interferon α and β (IFN-α/β) that induce inflammatory responses in tissues and increase cytotoxicities to the assaulting cells. These cytokines are responsible for providing innate immunity and play crucial roles in the control of tumor growth [56]. Macrophages are preeminent mononuclear phagocytes in immune system, killing invading pathogens as the first line of defense next to neutrophils. Apart from serving in defensive system, macrophages are involve in tissue repair, tissue development, and NME homeostasis [57]. In addition, macrophages also participate in vasculogenesis, angiogenesis, and maintenance of mammary stem cells [58, 59]. Their survival and proliferation are regulated by colony stimulating factor 1 (CSF1) [60]. Macrophages can be recruited by CSF1 and other tumor chemoattractants such as CCL2, VEGFA, and semaphoring 3A (SEMA3A) in developing TME [61]. Tumor-associated macrophages (TAMs) often interact with adipocytes in tissue stroma for cancer development and progression. TAMs phagocytose adjacent dead adipocytes and establish inflammatory foci known as crown-like structures (CLS) [62, 63]. Highly diverse population of macrophages or monocytes, generally known as myeloid cells, are terminally differentiated macrophages or dendritic cells (DC). Since these myeloid cells are responsible for suppression of various types of immune response, they are defined as myeloid-derived suppressor cells (MDSCs) [64]. MDSCs usually target T cells through suppressor factors like arginase (ARG1), TGF-β, IL-10, inducible nitric oxide synthase (iNOS), and cyclooxygenase-2 (COX2) [64].

### In vitro native microenvironment based on cells

Normal tissue stroma contains different types of cells such as residential specific cells, migrating cells, fibroblasts, immune cells, adipocytes and endothelial cells as described above (Fig. 1). Their spatial arrangements and communications are tissue-specific. NME transforms to TME in the presence of tumor cells and their associated stromal cells (Fig. 1). Mimicking native NME or TME in vitro based on the cell types within specific tissue matrices is critical for biologically and clinically relevant cancer studies and tissue engineering. Hence, it is fundamental to establish in vitro co-culture systems that are able to provide microenvironments highly resembling native tissue ECM at structural, mechanical and biochemical levels for different types of cells. Overall, co-culture using two different cell types becomes more common nowadays than before and contributes to our understanding about intricate cell-cell interactions and signaling mechanisms. For instance, co-culture of primary human mammary fibroblast and breast cancer MCF-7 cells revealed intercellular communications that were only possible to be observed in the presence of the secreted biomolecules such as IL-6, prostaglandin E2 (PGE2), and IL-6sR from the cancer cells [65]. Additionally, more complex co-cultures with multiple cell types have been reported with encouraging outcomes. For example, endothelial cells, fibroblasts and bone marrow stromal cells were used for new bone formation [66], and hepatocytes, fibroblasts and endothelial cells were applied in the study of liver tissue generation containing neocapillaries [67]. However, co-cultures with mixed cell populations in tissue-mimicking environments are bound to face certain technical challenges, such as the requirement of different or specific GFs and additional supplements for different types of cells in a same co-culture system, control of cell orientation and distribution, tools used to assess complex cell-cell interactions, and the coordination of the different parts of the assembled network for overall functions of the in vitro culture. Cultural conditions need to be formulated in a way to promote optimal survival and growth of all types of cells involved in forging the in vitro stroma of the co-culture, with a control over non-desired stimulation or inhibition of the different types cells in the system.

### ECM for native microenvironment

**Fig. 2:**
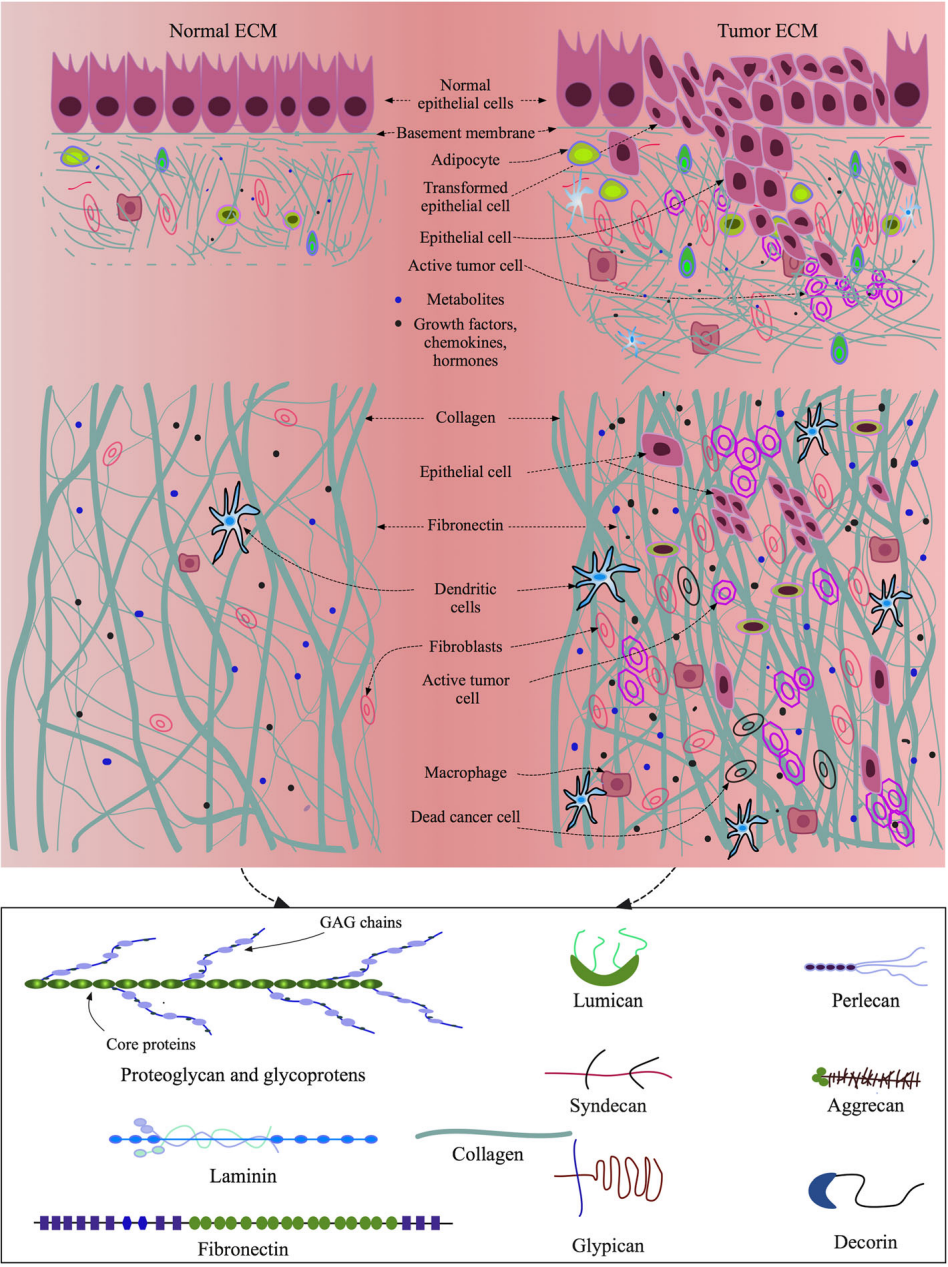
Normal and tumor ECM components and organizations in epithelial tissues. Native tissue ECM is modified by the activities of the cells living within it. While normal epithelial cells are isolated from connective tissue ECM by BM and barely apply modifications to the surrounding matrix, invasive epithelial cancer cells breakdown BM and aggressively remodel the surrounding ECM to build up TME, which harbors multi-lineage cells, newly produced ECM proteins to reinforce ECM structures, and enrich biomolecules in favor of tumor progression

The physically and physiologically active extracellular micro-areas surrounding tissue cells are organized by a protein meshwork, the ECM, which is another essential part of the microenvironment for the cells (Fig. 2). ECM not only acts as a structural scaffolding support for the cells but also provides mechanical and signaling guidance for their adhesion, distribution, proliferation, differentiation, and migration. Tensile and elastic strengths of ECM mediate cell-cell and cell-ECM interactions. Cells within ECM in turn modify the matrix by depositing additional or degrading existing ECM and secreting biomolecules that are needed for their optimal survival, growth, and other biological activities in responses to environmental changes or intrinsic mutations. Fibroblasts are believed to be the chief stewards for collagen deposition in mammary tissues [68]. When tumor arises in a breast, TAF generally deposits more collagen into ECM [69–71], which is comparably stiffer than the ECM under normal conditions. However, it was recently reported that TAF in invasive breast cancers had attenuated collagen generation [72]. In contrast, epithelial cancer cells but not TAFs were observed to produce collagen within tumor ECM [73, 74]. Moreover, macrophages was shown to promote collagen fibrillogenesis during mammary gland development [75], and TAMs can regulate fibroblasts to produce enzymes and inhibitors that mediate ECM degradation in tumors [76]. Furthermore, ECM cross-linkers such as transglutaminase and lysyl oxidase (LOX) present within TME participate in tumor ECM modifications by cross-linking the newly generated collagen fibers and other ECM proteins. The local tension change in a tumor and its surrounding tissues is a key mechanical factor regulating spatial cell migration and cancer progression [77, 78]. The presence of GFs, chemokines, angiogenic molecules, and MMPs in ECM provide additional stimuli to the intracellular and intercellular signaling networks that mediate the process of cancer development. Other proteases such as cathepsins and cysteine proteases are also highly expressed in TME. They suppress blood clotting mechanism by activating heparanase, thereby aiding angiogenesis and metastasis.

Recent advances in in vivo-mimicking tissue culture systems demonstrated the advantages of applying native ECM in biomedical research and bioengineering [7, 69, 79–82]. Yet, synthetic materials remain to be a robust source of ECM-mimicry in the field, depending on specific applications of the materials. Overall, both synthetic and native materials should provide not only structural, mechanical, and biochemical supports to cells cultured within the scaffolding materials but also optimally support cell-cell and cell-matrix interactions through native-mimicking signaling events. Hence, ECM is one of the most important components of microenvironment under both in vitro and in vivo conditions for the homeostasis, growth, and repair of tissues [83].

#### Synthetic ECM

The materials that are bioinert, biodegradable, biocompatible, and hydrophilic in nature are used for preparation of synthetic hydrogels. Hydrogels can be further converted to elastic topographical materials, generally known as synthetic ECM, after cross-linking of their polymers. Biophysical and biochemical cues of hydrogels can be spatially and temporally tuned to mimic native ECM for cellular activities such as adhesion, proliferation, and migration [84]. For example, hydrogels from poly(ethylene glycol) (PEG), poly(2-hydroxyethyl methacrylate) (pHEMA), Poly(ethylene glycol) diacrylate (PEG diacrylate), and poly(vinyl alcohol) (PVA or PVOH) can be prepared to structurally and mechanically mimic the physical aspects of tissue ECM. These synthetic polymeric hydrogels are often used as matrices in bioinks for tissue-mimicking printing. For example, PEG diacrylate and alginate blend serves as robust bioink for fabrication of tissue constructs as demonstrated by Rutz et al. [85] and Hong et al. [86].

PEG is able to polymerize under cytocompatible conditions through numerous reactions, such as Michael addition, chain polymerization, azide alkyne cycloaddition and thiol-ene. Besides, it can be functionalized by modifying its terminal hydroxyl residue with certain reactive groups, such as alkenes, alkyenes, thiols, N-hydroxysuccinimide (NHS) esters, maleimides and azides [87, 88]. Cell adhesion property can be enhanced by introduction of Arg-Gly-Asp (RGD) peptide sequences in PEG with NHS ester on one end and acrylate functional group on the other side [89, 90]. In addition to RGDs, other peptides and different ECM components can be incorporated into PEG and other biomaterials to maintain self-renewal and differentiation of stem cells. For instance, incorporation of vitronectin-derived heparin-binding peptide I (GKKQRFRHRNRKG) into polyacrylamide hydrogel facilitates the interaction of ECM with cell surface glycans, and addition of glycosaminoglycan-binding peptides supports self-renewal of stem cells [91, 92]. Likewise, hydrogels can be fabricated for a specific ECM function. For example, heparin-decorated Hyaluronic acid (HA) hydrogel was used to release bone morphogenetic protein-2 (BMP-2) for chondrogenic differentiation of murine mesenchymal stem cells [93]; addition of phosphate functional group into mineralized matrices or PEG hydrogel induced osteogenic differentiation of human mesenchymal stem cells (hMSCs) [94, 95] while inclusion of t-butyl moiety in the PEG gel promoted adipogenic differentiation [95]; mineralization of PEG hydrogels modified with varying lengths of anionic pendant side chains terminating with carboxyl groups was used in bone-mimetic composite material fabrication [96].

In addition to chemical modifications, elasticity and stiffness of hydrogels are key factors that play vital roles in cell differentiation. HMSCs grown on hydrogels at low elasticity (elastic modulus 0.1–1 kPa) undergo neurogenesis, while on stiffer hydrogels (elastic modulus 8–17 kPa) are routed to myogenesis and tougher hydrogels (elastic modulus 25–40 kPa) to osteogenesis [97]. Interestingly, hMSCs osteogenic differentiation can be triggered by hydrogels with early stiffening and adipogenic differentiation with late stiffening [97].

Moreover, hMSCs grown on hydrogel with high traction stresses differentiate into osteogenic lineage and undergo adipogenic differentiation on low traction gel [98]. Furthermore, hydrogel hydrophobicity influences cellular organization such as rosette-like clusters that help maintain the cellular morphology and promote differentiation of hMSCs [99]. Certain degree of hydrophobicity of hydrogel is required to maintain its adhesiveness that is necessary for cell attachment, proliferation and migration [99, 100]. A smart surface of hydrogel is therefore critical to allow cell attachment and growth. Hydrogels like poly(N-isopropylacrylamide) (pNIPAm), exhibiting lower critical solution temperatures (LCST), are generally used for the preparation of smart surfaces [101]. PNIPAm is hydrophilic at temperature below 37 °C, but becomes hydrophobic at 37 °C that enables nonspecific protein interaction for cell attachment. Some synthetic biomaterials like polycarpolactone (PCL) is entirely hydrophobic in nature and difficult to grow cells for tissue engineering and biomedical studies, while some hydrophilic biomaterials such as silk fibroin, aloe vera, and curcumin can be added to PCL to make smart surfaces for cell attachment [102]. PCL can also be coated with cell-laden ECM, alginate or decellularized ECM, making it more hydrophilic and native-mimicking for cells [103, 104]. Furthermore, PCL is one of the most widely used synthetic ECM in various formats of scaffolds for cancer studies because of its slow degradation kinetics and biocompatibility, supporting TME that contain cancer cells [105–107].

Another most abundantly used synthetic ECM is poly(lactic-co-glycolic acid) (PLGA) for both tissue engineering and cancer studies. PLGA is biodegradable in the body, releasing byproducts, lactic acid and glycolic acid in the presence of water [107, 108]. Both byproducts enter into various metabolic pathways in the body under normal physiological conditions. Some other polymers like poly(ethylene) (PE), poly(propylene) (PP), poly(vinyl chloride) (PVC), poly(dimethyl silane) (PDMS), poly(methacrylate) (PMMA), pHEMA, poly(ethylene terephthalate) (PET, dacron), poly-L-lactic acide (PLLA), poly-D- Lactic acide (PDLA), polydioxanome (PDO), polyether ether ketone (PEEK), polyether sulfonate (PES), polyamide nylon, poly(vinlypyrrolidone) (PVP), poly(styrene-b-isobutylene-b-styrene) (SIBS), ultrahigh molecular weight PE (UHMWPE), and polyurethane can be use in tissue engineering, suturing and other material engineering applications [109]. Apparently, the versatile synthetic polymers are rich sources for tissue engineering in addition to their applications in mimicking physiological tissue environments such as TME [110].

#### Native ECM

Chitosan is derived from partial deacetylation of chitin, which contains at least 60% of D-glucosamine residues [111]. Commercial chitosan is usually extracted from the chitin of crustaceans and fungal mycelia. The presence of protonable amino groups in chitosan is a peculiar property of the material. For example, a negatively charged sialic acid provides the mucoadhesion property of chitosan [112] and a positively charged amino acid in chitosan backbone endows its hemostatic activity and interaction with the negatively charged cell membrane, thereby helping reorganization of tight junction and membrane proteins and enhancing chitosan permeability [113]. The antimicrobial, polycationic and biodegradable natures of chitosan support its biomedical applications in different formats like hydrogels, films, sponges and 3D scaffolds [114–117]. It was also shown that chitosan promoted cancer progression in TME by binding to CSCs via CD44 receptors and activating both canonical and non-canonical signaling pathways [118].

Silk fibroin, a macromolecular protein polymer secreted by silkworm (*Bombyx mori*) larvae, is biocompatible and has been applied for tissue engineering of bone, cartilage, ligament and tendon, skin tissue, blood vessel, liver, spinal cord, trachea, bladder, and ocular tissues. Silk fibroin with or without inducing factors like hydroxyapatite and bone morphogenic proteins (BMPs) is extensively used for in vivo neobone formation together with different kinds of stem cells such as human osteoblasts, mesenchymal stem cells (MSCs), and MC3T3-E1 cells (osteoblast-like cell line) [119]. The protein polymer can be blended with chitosan for bone, cartilage, ligament and tendon tissue engineering. Skin tissues have been regenerated in rats after implantation of silk fibroin alone or blended with alginate, chitin, or collagen in the presence of human oral or epithelial keratinocytes and fibroblasts [120–123]. Accumulating data have shown that silk fibroin supports cultures of almost all types of stem cells that are specific for particular tissue regeneration in NME [124–126]. Enzymatically cross-linked silk fibroin can be used as bioink in tissue engineering such as musculoskeletal tissue regeneration for personalized implant and for versatile organ printing with structural stability and reliable biocompatibility that can be achieved by blending in methacrylate and fabricating with the digital light processing (DLP) bioprinting method [127, 128].

Furthermore, silk fibroin has been used to reconstitute TME and culture breast cancer cells and fibroblasts in vitro, where fibroblast-cancer cell as well as cell-ECM interactions that are important parameters of cancer progression were observed [129].

Starch has been used in tissue, particularly bone, engineering as scaffold, bone cement, or blended form to facilitate collagen deposition for neobone formation [130, 131]. The topography of starch-based scaffold can be modified by coating with adhesive proteins such as plasma proteins to enhance better attachment of cells to the scaffold surface and facilitate the growth of endothelial cells [132]. In combination with PCL, starch was also used in 3D rapid prototyping of layered hierarchical structures for hard tissue engineering [133]. Nanofibrous mats prepared by electrospinning of starch and polyvinyl alcohol (PVOH) support skin regeneration [134]. Starch so far has not been reported for the use of maintaining TME in tumor modeling.

Alginate is one of the most widely used biomaterials in tissue engineering, which is available in various formats like hydrogels, microspheres, microcapsules, sponges, foams, and 3D porous scaffolds. Alginate gel forms through polymerization of its components α-L-guluronic acid and β-D-mannuronic acid that increase the flexibilities of the polymers and enhance the capacities of the gel to trap water, cells and other molecules, representing bioink properties [135]. Alginate-based bioinks were used to print vessel like constructs and demonstrated excellent properties in supporting cell viability during and after a coaxial bioprinting process [136, 137]. Alginate can be blended with other bioprintable materials such as gelatin, hydroxyapatite, PCL for tissue engineering [138, 139]. Since alginate is composed of repeating units of α-L-guluronic acid and β-D-mannuronic acid monomers and lack of bioactive ligands necessary for cell-matrix interactions, RGD is often chemically coupled to alginate using water-soluble carbodiimide [140, 141]. RGD-modified alginate promotes cell attachment, proliferation, differentiation, and migration. Based on this functionality, RGD-alginate blended with various supporting factors and stem cells can be used in tissue engineering [142]. Alginate allows sustained release of entrapped GFs to support cell proliferation, neovascular formation, and delivery of entrapped cells to target sites. Alginate bioinks are capable of modulating release and spread of cells without affecting the integrity of alginate lattice structures for tissue regeneration in NME [143, 144]. Blending other biodegradable polymers such as PLLA, chitosan, gelatin, and PLGA into alginate has been used to transport molecules through epithelia and mucosa in various forms of microspheres or nanoparticles. With various modifications, alginate has also been applied in wound healing, cartilage repair, and bone regeneration. Alginate-based hydrogel, on the other hand, was used to form tumor spheroids in microfluidic culture systems in an attempt to mimic solid tumors [145]; to blend with other biocompatible biomaterials such as gelatin to make composite hydrogel and with tumor cells and TAFs for in vitro tumor models to study cell-cell interactions and mechanisms of tumorigenesis [146]; to mimic TME in 3D cultures for angiogenesis with the engagement of cancer cells, VEGF, and integrin [147].

Cellulose is a biopolymer polysaccharide isolated from bacteria or plants. Though being a natural extracellular matrix, cellulose is a very slowly degradable biomaterial when implanted into human tissues owing to the lack of required enzyme to digest it. Breakdown of cellulose is only possible through hydrolysis of glycosidic bonds and oxidation of the glucopyranose rings. Oxidized cellulose has been used as anti-adhesive tapes for wound healing. Oxidation of cellulose induces conversion of the glucose residues to glucuronic acid (with –COOH groups), which acts as the binding site for various molecules such as arginine, chitosan, and GFs, a mechanism that is useful for tissue engineering [148]. The antimicrobial properties of oxidized cellulose can be used for tissue repair. Six-carboxycellulose with low percentage of –COOH groups supports differentiation and maturation of stem cells rather than their proliferation [148]. A range of nanomaterials such as calcium carbonate, titanium oxide, and silicon carbide have been implemented to mix with cellulose to improve the resistance and stability of cellulose [148]. Cellulose has been minimally modified for the purpose of cell attachment in the absence of ligands or proteins by means of introduction of positive charge with 2,2,6,6-tetramethylpipiridine 1-oxyl or negative charge with sodium bromide, without altering its mechanical properties [149]. Cartilage repair, bone regeneration, and functional cardiac constructs have been tested using cellulose as 3D scaffolding hydrogels [150–152]. At last but not at least, cellulose scaffold was also applied in studies of human cancers, such as breast cancer, and found to be supportive for tumor formation [153].

Gelatin is a natural protein derived from partial hydrolysis of collagen. Though gelatin is mechanically weak, it is applied in tissue engineering after being stabilized and stiffened by various cross-linking methods, most commonly by photo cross-linking [154]. Photo cross-linkable gelatin with incorporation of furfuryl isocyanate (gelatin-FI) or furfurylamine (gelatin-FA) has been used as a dental pulp capping material and is a promising scaffold for osteochondral repair [155, 156]. Addition of BMP into gelatin-FA hydrogel further enhances articular cartilage and subchondral bone repair with more expression of osteochondrogenic factors like col1a1, col2a1, SOX9, and aggrecan [156]. Gelatin blended with other biomaterials such as calcium phosphate ceramics, alginate and chitosan has improved mechanical properties in scaffolding. For engineering of cardiac and nerve tissues, gelatin was developed into functional scaffolds with the addition of conductive molecules like polyaniline and carbon-based synthetic polymers [157]. Hybrid hydrogel made up of gelatin and chitosan was generated to support the formation of in vitro tumoroid, which expressed genes such as p21, integrin αV, N-cadherin, vimentin, CK-18 and β-catenin that are involved in tumor growth in TME similar to those in native tumors [158].

Hyaluronic acid (HA) is a glycosaminoglycan that is commonly found in native ECM of cartilages and connective tissues. It is often used as scaffold for tissue regeneration, wound healing and drug delivery or as bioink for 3D tissue constructs [159, 160]. Its use has been expanded to blending with other biomaterials for the regeneration of cardiovascular tissue, brain, cornea, lung and skin [161]. Because of the low mechanical and slow gelation properties of HA, addition of cell-adhesive oligopeptides in HA hydrogel increases both mechanical and cell-adhesion properties of the gel [162]. HA bioink blended with gelatin was shown to maintain proliferation of human adipose stem cells and their differentiation into adipocytes in adipogenic culture medium [163]. Chemically modified HA, for example with methacrylate, has enhanced mechanical properties and can be used for various tissue engineering purposes [164, 165]. HA-based hydrogel also has long been used as ECM-mimicking cultural matrix in human cancer studies from basic 3D culture and induction of angiogenesis to tumor modeling and identification of cancer cell-secreted metabolites [166–168].

Collagens are triple helix proteins and the most studied biomaterial in tissue engineering and biomedical scaffolding. This is based on their presence in all types of connective tissues, distributed from soft tissues to hard tissues, as prominent and major fibrous protein components (25% of the total dry weigh of mammals) [169]. Collagen I, II, III, V and XI are among 29 distinct collagens that form collagen fibers. Collagen I is the most abundantly used collagen as a gold standard in tissue engineering. Common sources of collagen I for tissue engineering are skin or tendon from bovine or porcine and rat tail among others like fish, sponges, and jellyfish. Collagens are biodegradable by naturally existing enzymes, collagenases. This degradation mechanism is very useful in tissue engineering per se, and the byproducts of the degraded collagens and their peptide derivatives can further enhance tissue restoration by attracting fibroblasts, which are collagen-producing cells distributed throughout the body [170]. Cells adhere to collagens directly through receptors or indirectly through linkers such as fibronectin. Cell receptors, which have the ability to recognize specific peptide sequences within collagen fibers, are divided into four groups. The first group, glycoprotein VI for example, binds to collagen peptide sequence with GPO motif (Gly-Pro-Hyp); the second group contains integrin family and discoidin domain receptor 1 and 2 (DDR1 and DDR2); the third group is integrin-types capable of recognizing cryptic motifs; and the fourth group of other cell receptors directly binds non-collagenous domain of collagen [171]. Collagen blended with other biomaterials such as glycosaminoglycans (GAG), chitosan, or elastin, has been fabricated to enhance the mechanical properties of the scaffolding materials and increase their enzymatic resistance for improved tissue engineering [171, 172]. Collagen-based hydrogels and porous scaffolds in various formats, with or without cells and co-factors, have been applied for decades in studies of bone and cartilage repair, skin regeneration, cardiac tissue development, urinary bladder and ureter regeneration, wound dressing, and many other medical directions [173–177]. In addition to its tremendous roles in tissue engineering, collagen is broadly used in biomedical research especially in tumor microenvironment modeling that provides favorable stromal TME for cancer and stromal cells [178]. The orientation of collagen fibers not only directs tumor cell intravasation, but also participates the process of cancer metastasis [179]. Increased collagen I deposition in TME makes tumor ECM stiff, stimulating tumor growth by modulating a set of signaling events, such as shifting the balance from prolactin signaling (JAK2/STAT5) toward tumor progression signaling (ERK) [180]. Collagens such as VI and XIα1 were found to be involved in epithelial-mesenchymal transition, angiogenesis, and metastasis [181, 182].

Native tissues have been used to produce decellularized (or acellularized) ECM (dECM) matrices, which are able to provide structurally and mechanically supporting microenvironments almost identical to those within native tissues. Therefore, tissue-specific and biocompatible dECMs are very promising for tissue engineering and regenerative medicine, and can be applied without the limitations from shortages of donor organs or tissues. Additionally, the natural biochemical composition of a dECM is an advantageous property. With certain supplements such as GFs, dECM promotes spatial cell growth, tissue repair and modeling [183, 184]. Because of the structural, mechanical and compositional advantages, tissue-specific dECMs have been extracted from a variety of native tissues such as skeletal muscle, skin, urinary bladder, small intestine, brain, heart, blood vessels and other types of tissues to generate raw ECM, reconstituted porous scaffolds, and hydrogels for a broad range of applications in tissue engineering and biomedical research. For instance, reseeding of tissue-specific stem cells in dECM scaffolds has the potential to rejuvenate the scaffolds as functional tissue grafts [185]. In addition to the major types of collagens, dECM from animals or human has complex mixtures of other ECM proteins such as glycoproteins, proteoglycans, and certain minor ECM proteins [14, 186, 187]. Meanwhile, each type of tissue generally has a specific ECM composition and biochemical cues that affect cells at different levels, from attachment and growth to migration and death. Usually, a specific type of dECM is selected for the growth of a tissue-specific stem cell lineage. For example, adipose dECM is for growing adipose derived stem cells (ASCs) and/or MSCs and liver dECM for hepatocytes [188, 189]. Yet, nonspecific dECM can also be used for nonselective applications, for instance culturing ASCs in placental dECM for adipose tissue engineering [190]. Soft composite hydrogels consisting of dECM and other biomaterials like fibrin and chitosan have been used as bioinks, which contain desired stem cells and inducing factors, in soft tissue engineering [191, 192]. Moreover, composite scaffolds fabricated using dECM and synthetic polymers such as PCL or PLGA have hydrophilic and necessary mechanical properties for tissue engineering, especially hard tissue regeneration [193, 194]. The use of dECM in tumor modeling has substantially enhanced the efficacies of in vitro mimicry of TME and increased the capabilities of revealing the mechanisms of tumor formation and metastasis [14, 195]. Identification of cancer-driving genes has been greatly facilitated by using recellularization of dECM with cancer cells. For example, decellularized human colon matrix was used as scaffold to co-culture retrovirus-transfected primer human colonic epithelial cells (hCECs) with human fibroblasts, and revealed the cancer driving genes LATS2, ASXL2, CAMTA1, DDX20, FXR1, MITF, and PAX7 [196]. The invasiveness of human cancer cells was also studied using dECM that is otherwise difficult to be closely mimicked in other types of 3D cultures, which hardly recapitulate native-like microenvironments [14, 197].

### In vitro native microenvironment based on ECM

Native ECM is a dynamic and complex scaffolding framework that maintains physiological cues of cells living in tissues and supports organ development and repair [198]. The structure, mechanics, component, organization, orientation, and function of ECM as discussed above not only reflect the peculiar physical characteristics of each ECM type, but define specific functional properties required by the cells in their NME or TME (Fig. 2). Therefore, to some extent, an ECM microenvironmental niche forges the biological performance of the cells and guides their fate, the differentiation and self-renewal of stem cells for example. Stem cells, tissue specific cells and migratory cells, on the other hand, modify local ECM microenvironment to make it serve the best for cellular functionalities. Bidirectional communications between tissue-specific cells and ECM are thus vital for normal functions of tissues. Currently, most 3D cultures focus on optimizing mechanical and structural properties of scaffolding matrices for cells, with minimal integration of physiological and biochemical cues into the culture systems. For instance, collagen type I is a major component of most native tissue ECM, but collagen I alone is an incomplete matrix source for induction of complete cellular functionalities and phenotypes. In addition, the extraction, physiological parameters, and reconstitution conditions of collagen are sensitive aspects that may hamper the overall performances of collagen-derived matrices in 3D tissue cultures [199]. Even though hybrid or composite biomaterials have some additional strengths compared to an individual biomaterial in terms of structural and mechanical properties, biodegradability, stability, release of trapped GFs or other factors, they are still far from mimicking NME or TME. Under the current 3D culture status, dECM remains a competitive biomaterial that is able to provide native tissue-like microenvironment and overcome the shortcomings of synthetic polymers, single native ECM proteins, and composite or hybrid hydrogels. The use of dECM, therefore has been exponentially increased in tissue engineering, regenerative medicine, and cancer studies in recent years [107]. However, the protocols preparing dECM and its derivatives mostly, if not all, involve a variety of detergents, enzymes, acids, or bases that may potentially alter ECM protein structures, configurations, chemical or physical properties, which need to be addressed further. Therefore, challenges remain to mimic NME or TME at high fidelity using dECM, unless there come revolutionary methods of decellularization and ECM protein extraction from native tissues that can retain all the ECM proteins in their native conformational and functional states.

### In vitro microenvironment based on spheroid or organoid model

In addition to the ECM scaffolding methods described above, cell spheroid is a widely used model for 3D tissue culture, drug screening, and personalized medicine testing [200]. Spheroids are clusters of cells, which adhere to each other via desmosomes, adherens or tight junctions [201–203]. Molecular gradients, cell-cell and cell-ECM interactions are able to be established within spheroids in a way to deliver various signals and mechanical forces to the cells, influencing the viability, proliferation and differentiation of the cells [200, 204, 205]. A variety of cells including normal pluripotent, mesenchymal stem cells, endothelial cells, and cancer cells have been used to form multicellular spheroids (MCSs) for various biomedical studies and tissue engineering applications [206–209]. MCSs may form through loose aggregation of cells, direct cell-cell contact, cadherin accumulation at cell membrane and cadherin-cadherin binding [210–212]. During spheroid formation process in tissue culture, cells can secrete ECM proteins as initial scaffolding bed that also serves as part of their living microenvironment [213]. Although questions remain as for whether the cell-cell interactions and signaling mechanisms in MCSs are comparable to those in native tissues and whether the cell-generated ECM is sufficient to mimic the heterogeneous native ECM, MCSs are still impressive 3D microenvironment-providing tools to mimic in vivo pathophysiological conditions. Additionally, collagen- and alginate-based multicellular tumor spheroids (MCTS) have been generated and used in evaluating gene expression profiles, signaling pathways, tumor modeling, and drug delivery efficiencies [145, 214–216].

Organoid is an advanced form of multicellular construct where embryonic stem cells or induced pluripotent stem cells self-organize into 3D organ-like structures owing to the self-renewal and differentiation capabilities of the cells. A number of in vitro models have been reported to generate organoids from primary mesenchymal cells, pluripotent stem cells (embryonic or induced), and tissue or organ slices [217, 218]. Usually, different biomaterials are used as embedding matrices for successful organoid formation, for example, collagen I for small intestine [219, 220], silk-collagen for neural tissue [221], Matrigel for mammary [6], retinal primordium [222], lung [223] and intestine [224], and PEG for intestinal organoids [225]. Applying tumor organoid in cancer research is advantageous over 2D and many of the other current 3D culture models that use cancer cell lines or patient-derived xenografts (PDX) tumor samples expanded in mice. This is because accumulating genetic changes in multiple-passaged cancer cell lines make the cells no longer represent the original primary cancer cells, and human tumors regrown and passaged in animals carry both tissue and cellular genetic heterogeneities that are different from the primary human tumors [226], even without considering the involvement of immune systems in the animals. With the integration of additional microenvironmental factors at both ECM component and cell co-culture levels and application of smart scaffolding biomaterials, both cell spheroid and organoid models are rapidly advancing toward more close mimicry of native microenvironments.

### Biological factors of native microenvironment

**Fig. 3:**
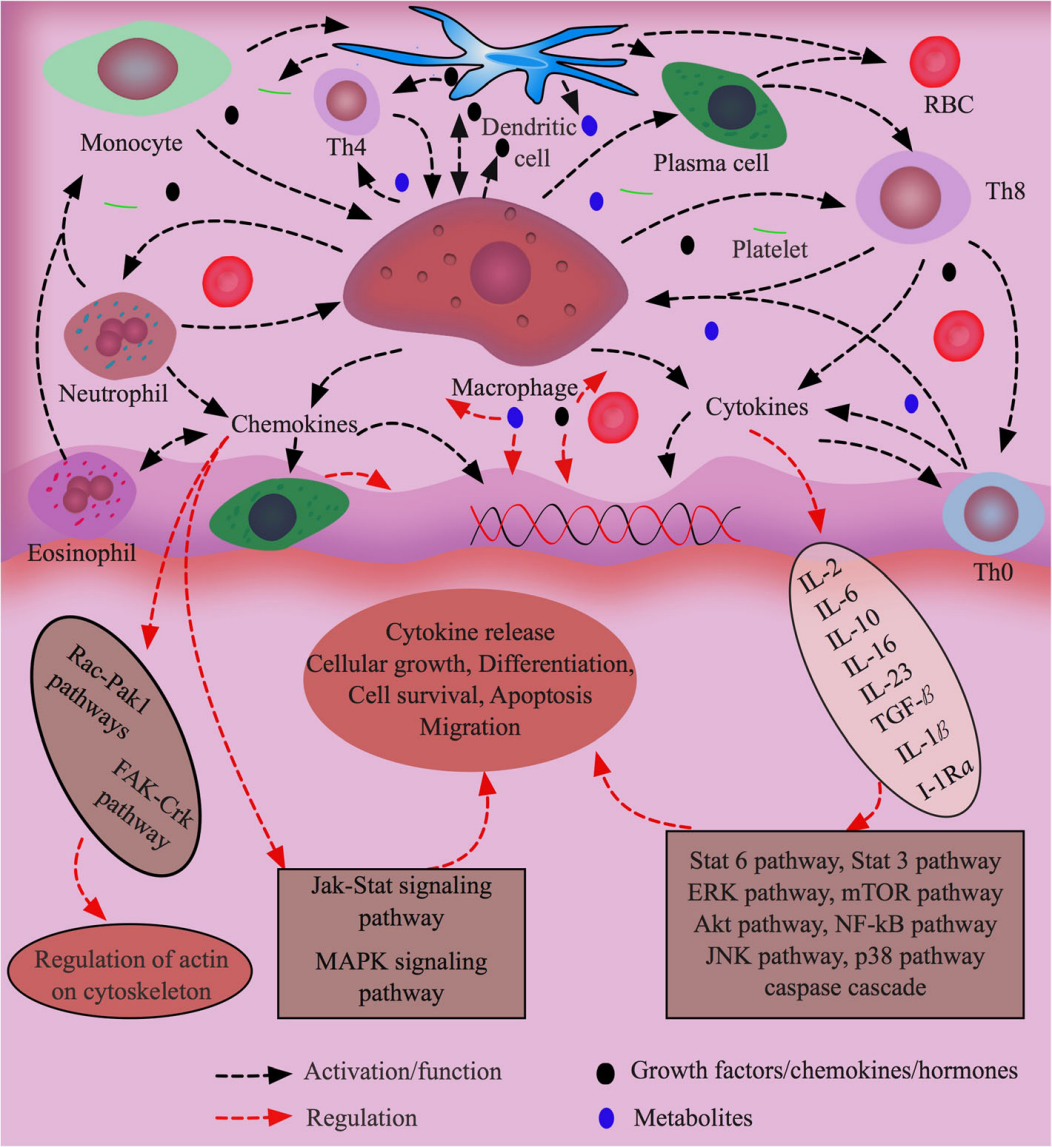
Biological factors and their roles in cell-cell and cell-matrix cross talks for tissue homeostasis. Various cytokines are released by activated immune cells such as monocytes, macrophages, dendritic cells and granulocytes. These cytokines activate or regulate other cells depending upon the microenvironmental conditions to maintain tissue homeostasis through various signaling pathways. In addition, growth factors, hormones and metabolites are essential factors to balance the tissue physiological conditions and functions. Besides defensive functions, immune cells are also actively involved in tissue growth, repair and blood vessel formation via their growth, differentiation, migration, and apoptosis and by sending signals to tissue specific cells, fibroblasts, platelets, red blood cells, and plasma cells. Tumor development is coupled with failure in the surveillance systems of immune cells, which are seized by tumor cells to produce high amount of active biological factors in tumor microenvironment to increase cell-cell and cell-matrix cross-talks for tumor progression

Chemokines, the largest family of cytokines, are highly potent factors in immunophysiological regulation of terminally differentiated and pluripotent stem cells for their chemotaxis activities (Fig. 3). They are typically divided into endogenous and exogenous soluble small proteins (8–14 kDa), and defined by the presence of four conserved cysteine residues. Generally, G protein-coupled receptors of tissue cells get activated by chemokines, inducing the cells to migrate through concentration gradient in a particular tissue where cells get accumulated for defense mechanisms [227]. There are approximately 50 endogenous chemokine ligands in mice and humans that are important to cellular and humoral immune responses and maintenance of tissue homeostasis. For example, CXCL1, CXCL2, CXCL3, CXCL5, CXCL6, CXCL7 and CXCL8 are involved in neutrophil trafficking, CXCL4 in coagulation, and CXCL9, CXCL10 and CXCL11 in the trafficking of natural killer cells, killer cells and helper cells by interacting with specific receptors like CCR or CXCR present on the plasma membrane of the cells [228]. Chemokines control not only the residence of immune cells in primary lymphoid organs, but their localization in secondary, tertiary lymphoid and periphery organs [229]. Neutrophils, Eosinophils, basophils, mast cells, monocytes, dendritic cells, lymphocytes, regulatory T cells, innate lymphocytes and resident immune cells are all directed and activated by chemokines at different levels for proper responses to antigens depending on defensing mechanisms. Moreover, cytokines are used to differentiate macrophages to dendritic cells, which express significantly more CD56, CD80, CD86, MHC class I and IL-10 compared to Monocyte-derived dendritic cells [230]. CXCL12 produced from bone marrow stromal cells in NME attract lymphocytes, monocytes and CD34^+^ hematopoietic precursor cells expressing chemokine receptor CXCR4 [231]. Interestingly, CXCL12 is also expressed by cancer cells in TME and, in coordination with CXCR4, regulates the migration of the cancer cells for metastasis [232].

T cells grown on 3D scaffold express various chemokine receptors such as CXCR1 to CXCR5 and CCR1 to CCR3, CCR5 and CCR6. Ivanoff and colleagues showed that chemokines interacted with 3D collagen I hydrogel substrata, and T cells exhibited migratory response to chemokine stimulation on the gel [233]. However, the chemokines failed to support infiltration of the cells into the collagen gel [233]. These observations were in contrast with the results that chemokine RANTES (CCL5), a ligand for CCR5, enhanced the generation of T cell focal adhesions and activated the cells through the FAK, ZAP-70 and paxillin protein complexes, and with that chemokines CCL2, CCL3, and CCL5 stimulated monocytes to express MMP-9 on 2D substrata [234, 235]. Since accumulating evidence support the concept that different levels or species of chemokines are expressed in 2D vs. 3D cultures [236], in depth studies on chemokine expression by immune cells grown on 3D synthetic or native tissue matrices will provide novel insights in whether different 3D culture models or materials induce similar chemokine production or chemokine receptor expression in the immune cells grown in the cultures.

GFs are polypeptides that stimulate cell proliferation, growth, and differentiation through binding to specific transmembrane receptors on target cells. Similar to chemokines, GFs are secreted by normal or transformed cells and distributed through plasma to their target sites. They can also act as cytokines or hormones through autocrine and paracrine mechanisms [237]. Excessive secretion of GFs by normal cells not only alters cellular responses that may result in diseases, but can lead to oncogenic transformation of the cells. Accumulating evidence have shown that the metabolic mechanisms and proliferation accelerated by GFs in normal cells are similar to those exhibited by neoplastic or cancer cells. PDGF, EGF, CSF-1, and TGF are four major GFs among others that have been studied broadly. PDGF is synthesized by megakaryocytes, stored in platelet granules, and released from platelets at injury sites once activated by thrombin. As a basic glycoprotein with 16 half-cysteine residues, PDGF is very active in its oxidized form and classified into PDGF-A, PDGF-B, PDGF-C, PDGF-D, and PDGF-F subtypes. PDGFs play vital roles during tissue and organ development as well as under normal physiological or cancerous conditions [238]. Similar to PDGF, EGF is a mitogenic polypeptide that promotes proliferation, survival, and differentiation of mammalian cells both in vivo and in vitro [239, 240]. On the other hand, EGF inhibits the stimulatory effects of certain hormones as a negative modulator [241, 242]. CSF-1 regulates the survival, proliferation, and differentiation of mononuclear phagocytes as well as promotes tissue growth and repair [243, 244]. Interestingly, CSF-1 and EGF exhibited contrasting effects in tooth development, where CSF-1 enhanced bone resorption by increasing the numbers of mononuclear cells and supported molar eruption while EGF accelerated osteogenesis and incisor eruption [245]. TGF- β is expressed and functions as an important signaling molecule in mammalian cells and a major player involved in regulating the composition and activation of ECM [246, 247]. It is generally activated and delivered or recruited to downstream signaling targets after ligand binding in ECM [248]. TGF-β promotes or inhibits cell proliferation and tissue growth based on the stimuli from surrounding environments and ECM perturbations so as to maintain tissue homeostasis [249].

Hormones are important biological factors (BFs) in the body for tissue maintenance and physiological functions through mediating the dynamic balance between cell proliferation and cell death. For example, androgens, prolactin, glucocorticoids, and estrogen stimulate epithelial cell proliferations and tissue growth of prostate gland, mammary gland, ovary and uterus, respectively, whereas corticosteroids and glucocorticoids promote apoptosis of thymus gland and bone, respectively [250]. Moreover, effects of hormones on cells depend on the concentrations of hormones since the responses of normal tissues are noticed only when hormones are in physiological range of concentrations. Dysregulation of concentration or presence of hormones in local tissues or neighboring tissues through autocrine or paracrine mechanisms may lead to abnormal functions and phenotypes of tissue cells, which can be triggered for genetic alternations and transformations into cancerous cells [251, 252].

### In vitro native-mimicking microenvironment based on biological factors

BFs like chemokines, GFs, and hormones are imperative for the co-ordination of tissue cells within their living microenvironmental networks for their survival, growth, differentiation, and proliferation, (Fig. 3). Almost all native cells such as MSCs, fibroblast, epithelial cells, endothelial cells, myeloblasts, erythroblasts, megakaryoblasts, leukocytes and macrophages secret and release BFs into tissue microenvironments as part of the biological functions of the cells and as a mechanism to maintain tissue homeostasis [253]. Secretion of BFs from cells can be detected in in vitro culture systems by means of proliferation, cytotoxicity, chemotaxis, protein induction, and other types of assays [254, 255]. The profiles of BFs in different cultures may substantially vary depending on cell types, culture time, concentration and types of stimulants [256]. Additionally, intercellular communications effect intracellular signaling and release of BFs. Therefore, co-culturing the same or different types of cells in the presence or absence of genetic modifications of the cells for specific biologically phenotypes is sometimes preferred in many studies. Co-culture systems exist in two major types, direct and indirect, where two or more than two different types of cells are allowed to grow under their optimal cultural conditions. BFs release or suppression in direct and indirect co-cultures is different. For example, direct co-culture of ASCs and peripheral blood mononuclear cells (PBMCs) increased the release of IL-6, CXCL9, CXCL10, CCL2 and galectin-1 and decreased the secretion of IFN-γ, TNF-α and galectin-3 from the cells. In contrast, an opposite secretion pattern was observed in indirect co-cultures of ASCs and PBMCs [257]. Therefore, cautions need to be taken when planning co-culture experiments (direct vs. indirect) to identify cell-secreted biomolecules in culture environments no matter whether the culture systems are 2D or 3D and made of synthetic or natural materials. Although it is technically challenging to accommodate all the optimal conditions in co-culture systems, substantial progress has been made on mimicking native physiological conditions by introducing different ECM ingredients, cytokines, GFs, and hormones into spatial 3D culture systems that lend promise to establish advanced co-culture systems closely mimicking NME or TME and to study pathological changes within ECM highly resembling human disease conditions.

### Physiological conditions of native microenvironment

Physiological parameters like temperature, pH, oxygen (O_2_) and carbon dioxide (CO_2_) concentration, ions, energy supply, and waste removal play significant roles in tissue homeostasis, growth, and death. Detailed description of each of these parameters is beyond the scope of this review, and only their important properties relevant to this topic are discussed. Cells are highly sensitive to temperature, mostly become inactive in temperatures below 4 °C. Depending on the type and the nature of cells, some cells remain active in body temperatures below the regular 37 °C or above 42 °C of very high fever. Temperature controls cell functions through alterations in the types and amounts of intracellular chemicals [258]. Satellite cells from different origins have different sensitivities to temperature in terms of proliferation and differentiation. For example, pectoralis major muscle satellite cells were highly proliferative in vitro when temperature changed from 38 °C to 43 °C, whereas biceps femoris muscle cells displayed a different proliferative manner during the temperature shift and proliferated at a higher rate at 33 °C – 39 °C and a lower rate at 43 °C than the pectoralis major muscle satellite cells [259]. Similar to temperature, pH plays a fundamental role in cellular functions by regulating cell cycle and proliferation, and acts as a checkpoint control in various signaling pathways under normal and cancerous conditions [260, 261]. It has been shown that extracellular pH of cancer cells is slightly acidic (pH 6.2–6.9) than that of normal cells (pH 7.2–7.5). Acidic environment not only promotes cancer cell transcription of tumor-promoting factors such as VEGF, IL-8 and hypoxia-inducible factor (HIF-1) [262–264], but increases the expression of proteases like MMPs and cathepsins that facilitate migration of the cancer cells [265].

O_2_ is vital for most of living organisms and cells. Its concentration in a tissue is instructive to the metabolism of cells living within that tissue microenvironment. A well-organized vasculature in tissues enables delivery of O_2_ through red blood cells. In vitro cultures are mostly performed in 20% O_2_, supplemented with 5% CO_2_ and 75% nitrogen (N_2_) gas [266]. Neither does the oxygen concentration favor each type of cells in their local microenvironments, nor it represents endogenous oxygen tensions in various tissues. For example, while human lung alveoli have an air pressure of 110 mmHg (14.5% O_2_), the alveolar venous pO_2_ is 80–100 mmHg (~ 13% O_2_) and arterial pO_2_ is 40 mmHg (~ 5% O_2_). Brain cells have about 35 mmHg (~ 4.5% O_2_), superficial skin has 10 mmHg (~ 1.5% O_2_), deep skin has 35 mmHg (~ 4.5% O_2_), small intestine has 60 mmHg (7.5% O_2_), liver has 30–40 mmHg (4–5% O_2_), kidney medullary has 10–20 mmHg (1.3–2.5% O_2_), kidney cortex has 50 mmHg (6.3% O_2_), muscle has 25–30 mmHg (3.5–4%O_2_), bone marrow has 50–55 mmHg (6.3–7%O_2_) and cells has 10–20 mmHg (1.3–2.5% O_2_) [266–269]. Normally, highly active and proliferative cells, such as cancer cells, require more oxygen supply. However, it is known that fast-proliferating cancer cells within a solid tumor survive well with low oxygen pressure known as hypoxic microenvironment and undergo aerobic glycolysis (Warburg effect) or even a nonglycolytic route to obtain energy and byproducts for their growth [270]. It is of note that tumor tissues are heterogeneous with uneven O_2_ distribution. For example, mice normal muscle has pO_2_ around 26 mmHg (~ 3% O_2_), whereas mice melanoma has pO_2_ around 2 mmHg (< 0.3% O_2_) along with heterogeneous distribution of anoxic or hypoxic tissue areas [266]. Thus, optimizing oxygen concentrations during cell cultures are important for optimal performance of the cells and for preventing the cells from oxidative stress. Because of overall low oxygen tensions in human tissues, engineering approaches for tissue repair and regeneration have not been very successful as expected [271]. To address this challenge, various functional biomaterials, such as oxygen delivery biomaterials, oxygen generating biomaterials, and oxygen releasing biomaterials that provide oxygen and prevent cells from ischemic necrosis, have been developed [104, 272–274]. On the other hand, hypoxia can enhance mechanical properties of engineered tissues as well as increase angiogenesis and deposition of specific ECM components in cancers.[275, 276].

Endogenous CO_2_ within human tissues is generally released during metabolic process as a by-product. Many studies demonstrated that CO_2_ binds with protein components of tissue cells and regulate various signaling and metabolic processes [277]. CO_2_ normally travels from its origin in tissues to the lungs via blood circulation either in dissolved form or as carbonic acid after reacting with water or binding with hemoglobin as carboxy-hemoglobin [278]. Carbonic acid and bicarbonate are very important in local tissue environment, contributing to acid-base homeostasis as well as controlling many metabolic and signaling processes. CO_2_ has been found to be required for cell proliferation and growth. However, high concentrations of CO_2_ may have adverse consequences on cells and tissues by reducing cell proliferation and disturbing O_2_ utilization by the cells, thereby causing less ATP production without attributing to cell death [279].

### In vitro native-mimicking microenvironment based on physiological conditions

**Fig. 4:**
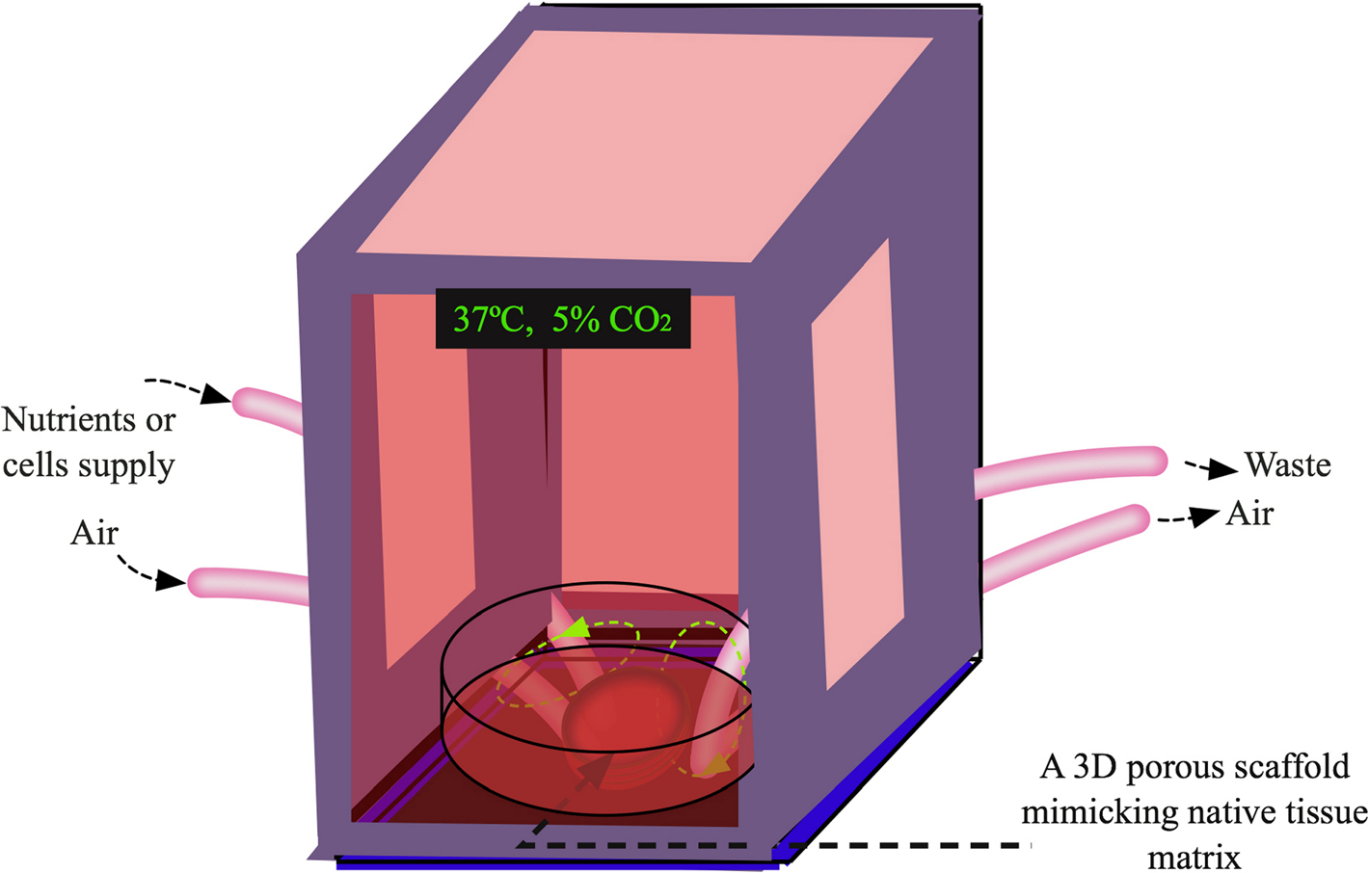
Dynamic spatial culture bioreactor with native-like microenvironment for in vitro tissue modeling and regeneration. The bioreactor is temperature, air, nutrient and cell supply controllable according to cell types and the nature of tissues. Specific tissue cells seeded on native ECM-mimicking porous scaffold prepared from biopolymers, native ECM proteins or decellularized ECM are cultured dynamically with constant rotation of the culture vessel to distribute gas and nutrient evenly under adjustable pneumatic pressure. Depending on the types of tissues to be cultured, the scaffold can be submerged in culture medium, suspended from the bottom, or as an air-liquid cultural design (part of the scaffold is exposed in the air over the media solution). Cultural media should be optimized for co-cultures of different types of cells

The physiological properties of human tissues are complex because of dynamic cellular functions and behaviors. Due to signaling changes within tissues or cells, activation and deactivation of cellular functions occur frequently, accompanied by taking in and secreting molecules from different cell populations of the same or different kinds. The full picture of human physiology has yet to be revealed. Indeed, 3D culture systems are able to provide spatial and architectural configurations to the cells. Yet, most of the current 3D culture models are devoid of heterogeneity of native tissue conditions, such as uneven distribution of nutrients and oxygen that creates hypoxic conditions in inner or central portion of scaffolds or hydrogels and zones of uneven cell growth. With the considerations of various physiological conditions such as ions, growth factors, cytokines, hormones, glucose and amino acids that are important for cell survival and growth in tissues, many attempts have been implemented to closely mimic tissue microenvironments for in vitro cell cultures. Encouragingly, different kinds of bioreactors have been developed for dynamic 3D cultures [280, 281]. These systems are equipped with oxygen and nutrient supplies as well as waste removal procedures for optimal cell proliferation, migration and differentiation (Fig. 4). Apparently, the perfusing dynamic 3D culture models are beyond the static culture states and will greatly facilitate biologically relevant studies.

## Conclusions and future perspectives

Our increasing understanding about tissue microenvironments at structural, mechanical and compositional levels has inspired researchers devising advanced scaffolds using various native or native-mimicking materials for biomedical and bioengineering spatial tissue cultures. This trend has been growing rapidly in recent years [107] that is benefited from refined methods for native tissue ECM extraction and protein purification, high resolution identifications of ECM components, broad availabilities of biomaterials and synthetic materials, close definitions of basic physical parameters of native ECM, vigorous development of stem cell technologies, advanced instrumental support, robust integration of computational modeling and mathematical algorithms. As a result of the advancement in the ECM modeling field, many biomedical studies have discovered novel molecules, functions, and phenotypes that are otherwise hard to be identified in 2D or non-biologically relevant 3D cultures [4, 282]. Meanwhile, bioengineers have combined the techniques of scaffold fabrication, bioink production and 3D printing for advanced tissue repair and regeneration [283, 284]. These encouraging progresses have greatly facilitated the development of novel systems to model pathophysiological conditions and approaches to treat human diseases.[8].

Meanwhile, the advancement in the field of biomaterial and scaffold fabrications has offered versatile tools for drug testing and delivery as well as for personalized medicine. Polysaccharides and their derivatives such as starch, chitosan and gums are most frequently used biopolymers in drug encapsulation and protection for effective drug delivery. Choice of polysaccharides in drug delivery is mainly due to their digestibility by specific enzymes in slower rate, providing longer time for target tissues to interact with the encapsulated drugs [285–287]. Biomaterials like alginate, gelatin, PEG, silk fibroin have also been used in drug delivery for many years. Formulation of nanoparticles such as micelles, liposomes, dendrimers and hydrogels with biopolymers are not only popularly implemented in drug delivery, but also commonly used in diagnostic applications [288]. Moreover, the advances in optimization and fabrication of biopolymers have brought them to broader applications as solubilizers, emulsifiers, gelling agents and viscosity enhancers for more efficient drug development and delivery [289, 290]. Recently, drugs are designed as bio-Nano-smart products for personalized medicine and gene therapy using advance biomaterials so as to guide the drugs to target sites, minimizing adverse side effects [291, 292]. Furthermore, blending of different types of biopolymers and mixing with treatment agents (e.g. oligonucleotides, gene products, compounds and small molecules) through multi-drug systems formulated and delivered as a single capsule, tablet or nanoparticle will certainly revolutionize therapeutic approaches for patients.

The capability of organoids to form patient-specific tissues makes the model very promising for tissue regeneration and cost-effective drug screening at personalized medicine levels [293, 294]. A combination of organoid models with optimized biopolymer or disease- and tissue-specific ECM scaffold systems under dynamic culturing environments represents a complex yet an ideal platform for future biomedical and bioengineering applications, which will form a new era of personalized medicine, precision therapy, effective drug development and delivery, functional artificial tissues and organs for the health of human.

## Abbreviations

TNF-α: Tumor necrotic factor
ASC: Adipose derived stem cell
ASXL2: Additional Sex Combs like 2
BFs: Biological factors
BM: Basement membrane
BMP: Bone morphogenic protein
CAMTA1: Calmodulin-binding transcription activator 1
CCL: Chemokine ligand
CCR: CC chemokine receptor
CD: Cluster of differentiation
CD4: Cluster of differentiation 4, surface protein on helper T cell
CD8: Cluster of differentiation 8, surface protein on cytotoxic memory T cells
CK: Cytokeratins
CLS: Crown-like structure
Colα1: Collagen alpha 1
COX2: Cyclooxygenase-2
CSCs: Cancer stem cells
CSF1: Colony stimulating factor 1
CTLA4: Cytotoxic T-lymphocyte antigen 4
CXCL: Chemokine (C-X-C motif) ligand
DC: Dendritic cell
DCIS: Ductal carcinoma in situ
DDX20: DEAD-box helicase 20
dECM: Decellularized extracellular matrix
DNA: Deoxyribonucleic acid
ECM: Extracellular matrix
EGF: Epidermal growth factor
EMT: Epithelial mesenchymal transition
FGF: Fibroblast growth factors
FOXP3: Forkhead box P3
FXR1: Fragile X-related 2
G-CSF: Granulocyte colony stimulating factor
GFs: Growth factors
GM-CSF: Granulocyte-macrophage colony stimulating factor
HA: Hyaluronic acid
hMSCs: Human mesenchymal stem cells
IFN- α: Interferon alpha
IFN- β: Interferon beta
IFN-y: Interferon gamma
IL: Interleukin
iNOS: Inducible nitric oxide
LATS2: Serine/threonine-protein kinase
LCST: Lower critical solution temperature
MCSs: Multicellular spheroids
MCTSs: Multicellular tumor spheroids
MDSCs: Myeloid-derived suppressor cells
MHC: Major histocompatibility complex
MITF: Mitochondria transcription factor
MMP: Matrix metalloproteinase
MSCs: Mesenchymal stem cells
NHS: N-hydroxysuccinimide
NK: Natural killer cell
NKT: Natural killer T cell
NME: Native microenvironment
PAX7: Paired box protein Pax-7
PCL: Polycaprolactone
PDGF: Platelet-derived growth factor
PDLA: Poly-D-Lactic acid
PDMS: Poly(dimethyl silane)
PDO: Polydioxanome
PE: Poly(ethylene)
PEEK: Polyether ether ketone
PEG: Poly(ehtylen glycol)
PES: Polyether sulfonate
PET: Poly(ethylene terephthalate)
pHEMA: Poly(2-hydroxyethyl methacrylate)
PLA: Poly(lactic acid)
PLGA: Poly(lactic-co-glycolic acid)
PLLA: Poly-L-lactic acid
PMMA: Poly(methacrylate)
pNIPAm: Poly(N-isopropylacrylamide)
PP: Poly(propylene)
PRP: Platelet-rich plasma
PVC: Poly(vinyl chloride)
PVOH: Polyvinyl alcohol
PVP: Poly(vinlypyrrolidone)
RGD: Arg-Gly-Asp
RME: Regenerative microenvironment
SEMA3A: Semaphoring 3A
SIBS: Poly(styrene-b-isobutylene-b-styrene)
SMC: Smooth muscle cell
TAF: Tumor associated fibroblast
TAM: Tumor associated macrophage
TGF-β: Transforming growth factor beta
TME: Tumor microenvironment
UHMPE: Ultrahigh molecular weight PE
VEGF: Vascular endothelial growth factor
VEGFA: Vascular endothelial growth factor A
VEGFC: Vascular endothelial growth factor C
VEGFD: Vascular endothelial growth factor D
